# Vara Deformity and Subluxed Humeral Heads: An Unusual Sign in Pseudohypoparathyroidism

**DOI:** 10.7759/cureus.53250

**Published:** 2024-01-30

**Authors:** Mason A Williams, Kharina Guruvadoo, Lena Naffaa

**Affiliations:** 1 Radiology, University of Central Florida College of Medicine, Orlando, USA; 2 Radiology, Nemours Children's Health System, Orlando, USA

**Keywords:** humeral head, parathyroid hormone (pth), slipped capital femoral epiphysis, bone resorption, vara deformity, pseudohypoparathyroidism

## Abstract

Pseudohypoparathyroidism is a rare disorder characterized by end-organ resistance to intact parathyroid hormone (PTH) and concomitant laboratory findings of hypocalcemia and hyperphosphatemia. Radiologic evidence of the disease may manifest as a variety of bone abnormalities. This case describes an 11-year-old female with a history of repaired bilateral slipped capital femoral epiphysis who presented with a limited range of motion of the bilateral upper extremities. Laboratory findings were consistent with pseudohypoparathyroidism. Radiographs revealed subchondral resorption of bilateral clavicular heads and multiple ribs and band lucencies of proximal humeral metaphyses, along with vara deformity and inferior subluxation of the humeral heads. This presentation adds to the spectrum of potential radiographic manifestations of pseudohypoparathyroidism.

## Introduction

Pseudohypoparathyroidism is a rare disorder characterized by end-organ resistance to intact parathyroid hormone (PTH). Elevated PTH, low calcium, and elevated phosphate levels are the hallmark laboratory findings. Imaging findings may reveal brachydactyly, ectopic ossifications, osteomalacia, and subchondral bone resorption [1]. Parathyroid hormone directly stimulates both osteoclasts and osteocytes, and it indirectly stimulates osteoclasts [2]. The pattern and intensity of the PTH signal determine the net effect on bone. The signal from chronically and continuously elevated PTH results in a net catabolic effect on bones [2]. In contrast, intermittent signals from PTH result in anabolic effects on bones [2]. Appropriate pattern and intensity of PTH signal are imperative for healthy bone formation and remodeling.

## Case presentation

**Figure 1:**
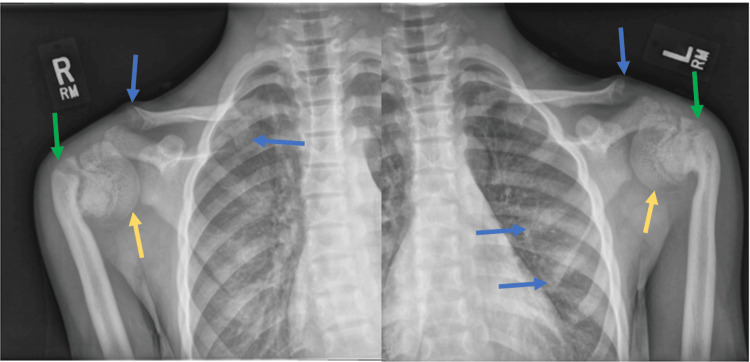
Plain films of bilateral shoulders Plain films of bilateral shoulders in pseudohypoparathyroidism demonstrating subchondral resorption of the ends of the clavicles and ribs (blue arrows), band lucencies of proximal humeral metaphyses with secondary vara deformity (green arrows), and inferior subluxation of the humeral heads (yellow arrows).

An 11-year-old female presented for a follow-up of surgical repair of bilateral slipped capital femoral epiphysis done in an outside facility and was found to have a limited range of motion of her bilateral upper extremities on examination. The physical exam was otherwise unremarkable. Plain film imaging of the shoulders revealed skeletal abnormalities suggestive of a high parathyroid hormone (PTH) effect on the bones. Radiographs showed subchondral resorption of the ends of the clavicles and ribs and band lucencies of the proximal humeral metaphyses with secondary vara deformity and inferior subluxation of humeral heads (Figure 1). Radiographs did not demonstrate osteosclerosis or subperiosteal bone resorption, as would be expected in primary hyperparathyroidism. There was also no evidence of the Rugger-Jersey spine, which appears as alternating bands of lucency and sclerosis that give the spine a striped appearance, as seen in secondary hyperparathyroidism. Radiographic appearance favored the diagnosis of pseudohypoparathyroidism. The diagnosis was confirmed by hypocalcemia (8.9 mg/dL), hyperphosphatemia (7.1 mg/dL), and elevated intact PTH (860 pg/mL), as seen in Table 1. The patient was placed on a regimen of calcitriol, calcium acetate, and magnesium oxide to normalize the laboratory values prior to any surgical corrective intervention and to prevent further complications such as pathologic fracture, avascular necrosis, and secondary permanent limb deformities. 

**Table 1:** Patient's laboratory values compared to reference ranges for age

	Patient Value	Reference Range
Calcium	8.9 mg/dL	9.1-10.5 mg/dL
Phosphate	7.1 mg/dL	3.3-5.1 mg/dL
Parathyroid hormone	860 pg/mL	15-65 pg/mL

## Discussion

Pseudohypoparathyroidism is caused by end-organ resistance to PTH and can be classified into several subtypes depending on the clinical presentation and type of PTH resistance. Type 1a pseudohypoparathyroidism, also known as Albright’s hereditary osteodystrophy, is the most common and is classically characterized by short stature, round face, brachydactyly, hyperparathyroid bone disease, and subcutaneous ossifications [3]. Type 1b, as seen in this patient, is a less common subtype that lacks these phenotypic features but still exhibits the effects of PTH resistance at the proximal renal tubules and high PTH effect on the bony skeleton. In both types 1a and 1b, resistance at the level of the kidneys leads to hypocalcemia and hyperphosphatemia, whereas the bone can remain sensitive to the resultant elevated PTH.

Parathyroid hormone has both anabolic and catabolic effects on the bones, and the net effect is based on the pattern and intensity of the PTH signal [2]. PTH upregulates the production of mRNA for receptor activators of nuclear factor-kappa B ligand (RANKL), which ultimately acts to increase osteoclast activity [2,4]. Continuous predominant osteoclast activity can inhibit normal ossification and bony maturation of the growth plates. In pseudohypoparathyroidism, the chronically elevated PTH signal is catabolic and results in bone resorption and impaired maturation. A combination of bony resorption at the end of bones and impaired development of the metaphyseal growth plates has been shown to lead to weakening and a propensity for secondary deformities [3,5,6]. It has previously been demonstrated in cases of slipped capital femoral epiphysis [3,5]. In this case, multifocal bony resorption and weakening allowed for vara deformity and slippage of the humeral heads.

The high PTH effect in pseudohypoparathyroidism may not cause subperiosteal bone resorption or osteosclerosis. However, this case did demonstrate subchondral bone resorption, which may be seen with any etiology of high PTH. The mixed features of the spectrum of diseases with high PTH can often lead to a delayed diagnosis among patients. PTH resistance, as seen in pseudohypoparathyroidism, should be treated with activated forms of vitamin D, which will increase the serum calcium level [7].

## Conclusions

Appropriate pattern and intensity of PTH signal are imperative for healthy bone formation and remodeling. The spectrum of parathyroid hormone disorders can be identified radiographically. Subchondral resorption of the bones is a telltale sign of high parathyroid activity on the bones. Subchondral resorption can lead to further deformity or joint malalignment.
